# Effect of Iron Oxide Nanoparticles and Amoxicillin on Bacterial Growth in the Presence of Dissolved Organic Carbon

**DOI:** 10.3390/biomedicines5030055

**Published:** 2017-09-08

**Authors:** Kelley M. Current, Niluka M. Dissanayake, Sherine O. Obare

**Affiliations:** Department of Chemistry, Western Michigan University, 1903 West Michigan Ave., Kalamazoo, MI 49008, USA; kelley.m.current@wmich.edu (K.M.C.); nilukamadhumi.m.dissanayake@wmich.edu (N.M.D.)

**Keywords:** engineered nanoparticles, iron oxide nanoparticles, emerging contaminants, amoxicillin, antibiotics, humic acid, bacteria

## Abstract

The impact of emerging contaminants in the presence of active pharmaceutical pollutants plays an important role in the persistence and activity of environmental bacteria. This manuscript focuses on the impact of amoxicillin functionalized iron oxide nanoparticles on bacterial growth, in the presence of dissolved organic carbon (humic acid). The impact of these emerging contaminants individually and collectively on the growth profiles of model gram positive and negative bacteria was tracked for 24 h. Results indicate exposure to subinhibitory concentrations of amoxicillin bound iron oxide nanoparticles, in the presence of humic acid, increase bacterial growth in *Pseudomonas aeruginosa* and *Staphylococcus aureus.* Accelerated bacterial growth was associated with an increase in iron ions, which have been shown to influence upregulation of cellular metabolism. Though iron oxide nanoparticles are often regarded as benign, this work demonstrates the distinguishable impact of amoxicillin bound iron oxide nanoparticles in the presence of dissolved organic carbon. The results indicate differential impacts of combined contaminants on bacterial growth, having potential implications for environmental and human health.

## 1. Introduction

The increased use of nanoparticles and their status as emerging contaminants has motivated researchers to investigate their impact on bacterial species, particularly with regard to antimicrobial activity. Among the most highly designed and utilized nanoparticles, iron oxide, titanium dioxide, zinc oxide, silica, and silver feature most prominently. In 2008, Lee et al. demonstrated the capacity of iron oxide nanoparticles to deactivate *Escherichia coli* [1]. In 2005, Morones et al. demonstrated diminished bacterial growth of *E. coli*, *Scrub typhus*, *Pseudomonas aeruginosa*, and *Vibrio cholera* upon exposure to silver nanoparticles [2]. In 2004, Sondi & Salopek-Sondi investigated silver nanoparticles, and demonstrated their bactericidal effects [3]. Furthermore, in 1993 the bactericidal effects of titanium dioxide nanoparticles were investigated as a potential technology for water purification [4]. Many researchers have continued to explore the potential applications and mechanistic causes of nanoparticles acting as biocides. While much research regarding the interaction of nanoparticles with bacteria has focused on antimicrobial activity, there are other outcomes that arise from nanoparticle-bacteria interactions that are yet to be understood. 

Environmental bacteria are ubiquitous, incredibly diverse, and play a crucial role in the cycling of elements within our environment. Environmental bacteria contribute to the cycling of carbon, nitrogen, sulfur, iron, and oxygen [5]. A study of nanoparticle-bacterial interactions in conjunction with common pollutants and naturally occurring compounds was conducted as a means of assessing the environmental impact of these entities, in conjunction with one another. Because environmental bacteria play a critical role in the maintenance and health of our ecosystems, and because environmentally transformed nanoparticles are known to induce differential impacts on biota, we show an investigation of: amoxicillin (Amox), iron oxide nanoparticles (IONPs), and amoxicillin functionalized IONPs (IONP-Amox) on bacteria, in the presence of dissolved organic carbon (DOC), on bacterial growth.

**Figure 1:**
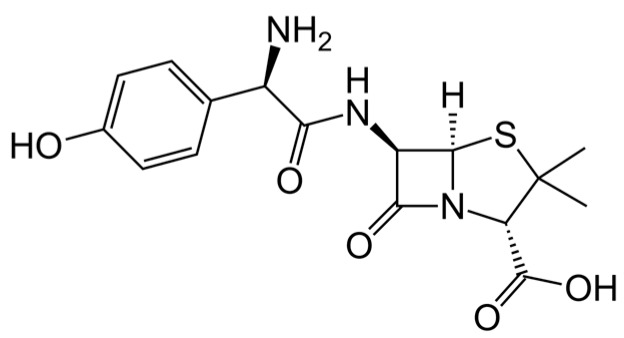
Chemical structure of amoxicillin (Amox).

Antibiotics of an anthropogenic origin have recently been shown to induce a variety of morphological and transcriptional changes in bacteria. These changes include increased virulence, altered gene transcription profiles, amplified horizontal gene transfer, and robust biofilm formation [6,7,8,9,10]. Amox (Figure 1), a β-lactam, was specifically selected for investigation because it is commonly prescribed within healthcare settings and is widely used in agriculture. According to a 2015 report by The Center for Disease Dynamics, Economics, and Policy group, the US is the world’s third largest consumer of healthcare antibiotics at 10,000 standard units (SUs), prescribed in 2010 [11]. Amox is one of the most commonly prescribed antibiotics on the market; in 2013, of the 48 million antibiotics prescribed, 6.7 million prescriptions contained Amox [11,12]. In addition to being widely utilized within healthcare settings, recent findings demonstrate that approximately 80–90% of an antibiotic dose is excreted in parent form, post consumption [13,14].

Agricultural utilization of antibiotics, including Amox, is also widespread [15]. More antibiotics are used on livestock than are used in all of humanity [11]. Interestingly, agricultural antibiotic use is common; however, reliable data regarding usage patterns (dose, frequency, and rational) have been poorly documented [15]. In agricultural settings, rationales for antibiotic usage includes the treatment of bacterial infections, incorporation into livestock feed as a prophylactic for the prevention of future bacterial infections, and as a growth promoter [11]. As global demand for meat products rise, so too does the application of agricultural antibiotics [11]. 

Like antibiotics, nanoparticles also represent a class of emerging contaminants impacting environmental bacteria. Nanoparticles are increasingly being utilized in consumer products including cosmetics, food packaging, therapeutics, drug delivery systems, diagnostics, and biosensors [16]. In accordance with their increased use, nanoparticles are entering the environment at increasing rates and represent a concerning class of emerging contaminants [16]. Like anthropogenic antibiotics, recent work has tied bacterial interaction with nanoparticles to altered behavior [9,17,18].

IONPs were specifically selected for study because they are used in a variety of settings and are widely regarded as innocuous. These nanoparticles are synthesized using a variety of facile, reproducible, and economically attractive routes, and often possess unique and functional properties. Because of their apparently benign nature and unique characteristics, researchers have investigated the potential to use IONPs in drug delivery systems, hyperthermia agents, magnetic resonance imaging contrast agents, and catalysts for environmental remediation [19,20,21,22,23,24,25]. 

Upon entering the environment, IONPs undergo a variety of chemical and physical changes, altering their surface and consequently their interactions with environmental species [26,27,28,29]. Nearby environmental chemical species are known to readily coat nanoparticle surfaces via surface ligand exchange. In an aqueous environment, the Fe and O atoms at the IONP surface are expected to adsorb OH^−^ and H^+^ ions. Because of their hydroxyl rich surface, IONPs are expected to bind Amox at the carboxylic acid moiety [30,31].

As the use of IONPs and antibiotics continues to increase, their environmental exposure is likely to also increase. Thus, it is important that studies not only focus on the effect of individual contaminants, but also on combinations of contaminants to best understand their collective environmental effects. The work described herein demonstrates the impact of combined contaminants on environmentally significant bacteria.

## 2. Experimental Section

### 2.1. Materials 

Iron (III) chloride hexahydrate (Alfa Aesar), iron (II) sulfate heptahydrate (Sigma Aldrich Company, Milwaukee, WI, USA), ammonium hydroxide (28–30%) ACS reagent grade (Sigma Aldrich Company), compressed nitrogen gas ultra-high purity (Airgas, Kalamazoo, MI, USA), humic acid technical grade (Sigma Aldrich Company), and Amox (>900 μg/mg) (Sigma Aldrich Company were purchased and used with no additional purification. Bacterial cultures were provided by Vivian Locke (Kalamazoo, MI, USA), Western Michigan University, Department of Biological Sciences Staff Member, and were grown using tryptic soy broth/agar (Sigma Aldrich Company). A 1X phosphate buffer solution (8.0 g sodium chloride, 0.2 g potassium chloride, 1.5 g disodium phosphate, 0.24 g potassium dihydrogen phosphate dissolved in 1 L of sterile deionized water) comprised of salts all purchased from the Sigma Aldrich Company was used. 

### 2.2. Iron Oxide Nanoparticle (IONP) Synthesis

IONPs were synthesized following modified literature procedures [32,33]. Briefly, a solution containing a 1:1 volume to volume ratio of 40 mM Fe^3+^: 40 mM Fe^2+^ solution (10 mL in total) was purged with N_2_ and placed under vigorous agitation. NH_4_OH (400 μL of 25% NH_4_OH and 40 mL of 18.5 Ω Milli-Q water) was added dropwise to the Fe^3+^/Fe^2+^ solution with continued agitation. Upon the addition of the basic solution, a color transition was observed (orange to dark brown). The solution was left under agitation for 60 min. IONPs were collected using a neodymium magnet, washed 3× with deionized water (100 mL aliquots), and dried for further characterization. 

### 2.3. Amoxicillin Functionalized Iron Oxide Nanoparticle (IONP-Amox) Synthesis

Two methods were used to functionalize Amox on IONPs. In the first method, IONPs and Amox were mixed for 24 h. These nanoparticles were synthesized (Supplementary Materials Figure S2); however, reproducibility was challenging. In this regard, a well-established literature procedure for IONP functionalization was adopted. Briefly, a co-precipitation method was employed where 40 mM Fe^3+^/40 mM Fe^2+^ solution (10 mL total volume) was purged with N_2_ and placed under vigorous stirring. To the Fe^3+^/Fe^2+^ solution, an NH_4_OH solution containing Amox (400 μL of 25% NH_4_OH, 40 mL of 18.5 Ω Milli-Q water, and Amox (0.01 g)) was added dropwise to the Fe^3+^/Fe^2+^ ion solution. As the Amox-containing NH_4_OH solution was added, a color transition was observed (orange to dark brown); the mixture was left under agitation for 60 min. IONP-Amox were collected via a neodymium magnet, washed 3× with deionized water (100 mL aliquots) to ensure the removal of unbound Amox, and dried for further characterization.

### 2.4. Nanoparticle Characterization: Transmission Electron Microscopy (TEM) & Thermogravimetric (TG) Analysis

IONPs and IONP-Amox were characterized by transmission electron microscopy (TEM) and thermogravimetric (TG) analysis. Nanoparticle TEM images were obtained by dropping IONP or IONP-Amox solution aliquots on 200 mesh Formvar/Carbon copper grids. The grids were dried and subsequently imaged using a JEOL JEM-1230 TEM at 80 kV transmission mode. Thermogravimetric (TG) analysis was performed using a TA Instrument (Model Number: 953001.901) by adding ~1 mg sample of IONP or IONP-Amox to a palladium pan. Each sample was heated at 10 °C/min from 22 °C to 600 °C under N_2_ (15 mL/min). 

### 2.5. Bacterial Culture Conditions

*Staphylococcus aureus* and *P. aeruginosa* cultures were seeded in sterile tryptic soy broth (TSB) (10 mL in 50 mL screw cap centrifuge tube) with single bacterial colonies. Once inoculated, all cultures were placed horizontally in a rotating incubator (37 °C) for 24 h. These cultures were utilized in all outlined experimental conditions; initial optical density (OD_600_) measurements began at ~0.200.

### 2.6. OD_600_ Time Series Study Outline

**Figure 2:**
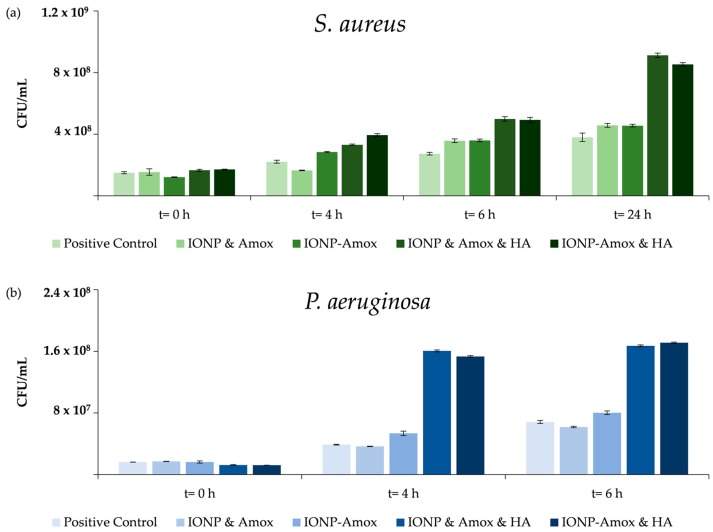
(**a**) *S. aureus* CFU/mL control condition values for time points 0, 4, 6, & 24 h. Error bars indicate ± SD; (**b**) *P. aeruginosa* CFU/mL control condition values for time points 0, 4, & 6 h. Error bars indicate ± SD. Both graphs indicating similar effects of IONP-Amox/IONP & Amox, and IONP-Amox & HA/IONP & Amox & HA (functionalized vs. unfunctionalized) on bacterial growth.

Appropriate amounts of IONP, Amox, IONP-Amox, and humic acid (HA) solutions (utilizing 1X phosphate buffer solution) were added to bacterial cultures and placed in 50 mL screw cap centrifuge tubes. When all conditions were fully constructed (at a total volume 5 mL), IONP and IONP-Amox were present at 0.2 μg/L, HA at 0.05 mg/L, and Amox at 1.5 ng/L. These nanoparticle concentrations represent predicted environmental nanoparticle concentrations, while the antibiotic concentration represents previously determined minimum inhibitory concentration (MIC) values [34,35]. Positive and negative controls were constructed and handled as experimental conditions. Positive controls contained culture, tryptic soy broth (TSB), and phosphate buffer solution but lacked IONP, Amox, IONP-Amox, and HA. Conversely, negative controls contained IONP, Amox, IONP-Amox, HA, phosphate buffer solution and TSB but lacked culture. Initial trials were carried out, demonstrating similar effects of IONP-Amox and IONP & Amox (functionalized vs. unfunctionalized) on bacterial growth, see Figure 2. The remainder of the paper focuses on Amox-functionalized IONPs. 

After construction, all conditions were placed in a rotating incubator (37 °C), maintained at pH 6.8, and sampled (3 × 100 μL subsamples) at designated time points. Subsamples were placed in polystyrene 96-well plates for optical density (OD_600_) measurement; such values correspond to the concentration of turbid bacterial cells and an increase represents increased bacterial concentration [3,36]. All experiments were conducted independently, in triplicate.

### 2.7. OD_600_ to CFU/mL Calibration Plot

A calibration plot comparing OD_600_ values to colony forming units per mL (CFU/mL) was constructed. *P. aeruginosa* and *S. aureus* cultures were grown for 12 h in a rotating incubator (37 °C). Each culture was serially diluted, yielding optical density values ranging from 0.200–0.900. Representative OD_600_ values were serially diluted (10^−3^ to 10^−7^), plated (60 mm sterile TSA petri dishes), and counted using standard procedures outlined by Collins et al. [37].

### 2.8. Statistical Analysis

Statistical analyses were carried out using Minitab (*v*. 17) with a repeated two-way ANOVA measure. Differentiation of statistically distinct mean values (α = 0.05) were determined via Tukey Multiple comparison tests. A detailed description of statistical groupings is provided in the supplementary information section (Table S1 and Figure S1). 

## 3. Results and Discussion

The work presented represents a model by which the environmental impacts of active pharmaceutical pollutants, in conjunction with nanoparticles, may be investigated. IONPs and Amox represent concerning emerging contaminants, and the impact of these contaminants, when present together, has yet to be investigated. The results outlined here demonstrate the impact of environmentally relevant IONPs and Amox concentrations on *P. aeruginosa* and *S. aureus* and are contextualized in terms of impact on bacterial growth and environmental effect.

### 3.1. TG Analysis and TEM Comparison of IONP and IONP-Amox 

**Figure 3:**
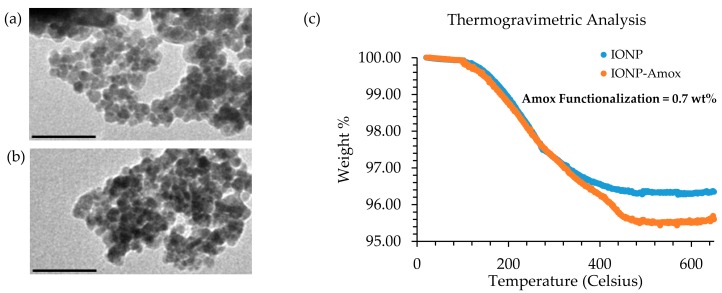
TEM images of: (**a**) IONP-Amox (scale bar = 100 nm); (**b**) IONP (scale bar = 100 nm); and (**c**) TG analysis data for IONP & IONP-Amox data.

Samples were analyzed by TEM and TG analysis to confirm IONP and IONP-Amox preparation. Samples were prepared for TEM analysis by drying 100 μL subsamples of each nanoparticle solution (IONP and IONP-Amox) on a 200 mesh Formvar/Carbon copper grid. Post desiccation, each grid was imaged using a JEOL JEM-1230 TEM set to 80 kV transmission mode. Resultant TEM images (Figure 3a,b) indicate the preparation of spherical monodisperse IONPs and IONP-Amox.

TG analysis was performed to confirm Amox functionalization of the IONP surface, as shown in Figure 3c. Approximately 1 mg of dried IONP and IONP-Amox was placed in a palladium pan and heated to 650 °C under N_2_. TG analysis of IONP-Amox was carried out to confirm Amox functionalization and to ensure stability [33]. Because Amox was present during IONP synthesis, the IONP & IONP-Amox curves overlap at lower temperatures, only at higher temperatures do differences become apparent, indicating that Amox is not removed easily.

To determine the individual and collective impact of IONPs, Amox, and IONP-Amox on *P. aeruginosa* and *S. aureus*, a series of controlled experiments were performed. *P. aeruginosa* and *S. aureus* cultures were combined with IONP (0.2 μg/L), Amox (1.5 ng/L), IONP-Amox (0.2 μg/L), and HA (0.05 mg/L).

Dissolved organic carbon (DOC) is present in nearly all ecosystems, and HA is an active component of DOC [38,39]. Furthermore, HA modifies colloids altering their fate, toxicity, and transport through the environment [27]. The influence of HA on nanoparticle-bacteria interactions within an environmental setting cannot be discounted. Previous work has demonstrated the sensitivity of bacteria and nanoparticles to HA; thus, HA was used to model the effects of DOC on IONP, Amox, and IONP-Amox interactions with bacteria. Fabrega et al. investigated the growth of *Pseudomonas fluorescens* cultures exposed to silver nanoparticles and HA, ultimately demonstrating HA’s protective effect in the presence of otherwise toxic silver nanoparticles [40]. HA has been shown to protect bacteria from diverse environmental stressors, including pollution, UV radiation, viral infection, and drought [41,42,43].

### 3.2. Impact of Amox Containing Paired Stressors

**Figure 4:**
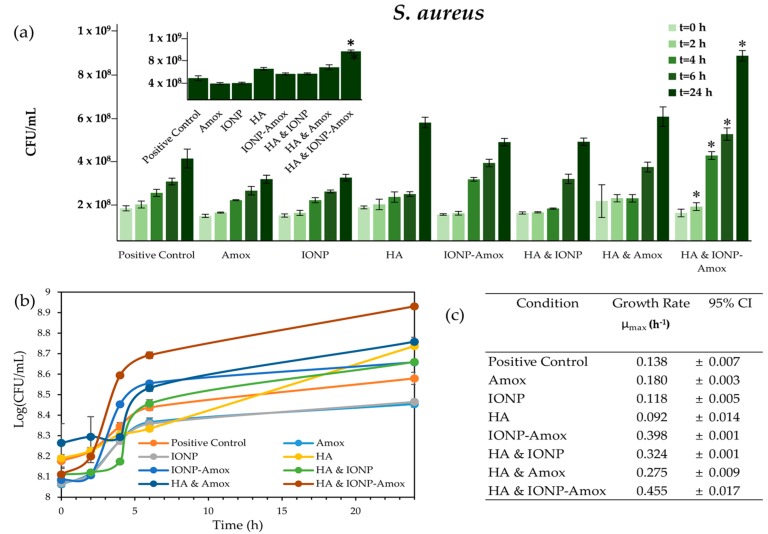
(**a**) *S. aureus* CFU/mL values for time points 0, 2, 4, 6, & 24 h. Error bars indicate ± SD, asterisk indicates statistical distinction (α = 0.05) from all other conditions at respective time points. Data indicated by an asterisk statistically differ from all other data within the respective time set; other statistical differences are described in the narrative. Inset graph represents *t = 24* h; (**b**) *S. aureus* growth profiles. Error bars indicate ± SD; (**c**) *S. aureus* maximum μ_max_ (h^−1^). 95% Confidence Intervals are indicated.

**Figure 5:**
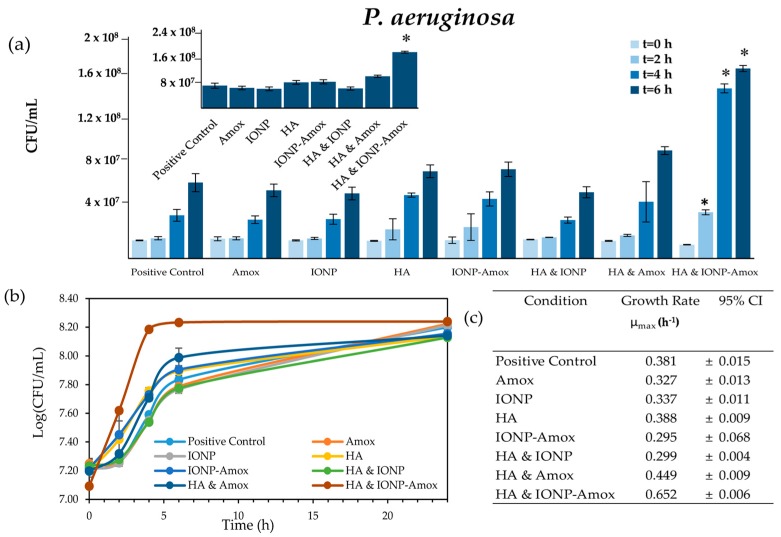
(**a**) *P. aeruginosa* CFU/mL values for time points 0, 2, 4, & 6 h. Error bars indicate ±SD, asterisk indicates statistical distinction (α = 0.05) from all other conditions at respective time points. Data indicated by an asterisk statistically differ from all other data within the respective time set; other statistical differences are described in the narrative. Inset graph represents *t* = 6 h; (**b**) *P. aeruginosa* growth profiles. Error bars indicate ± SD; (**c**) *P. aeruginosa* μ_max_ (h^−1^). 95% Confidence Interval are indicated.

Nanoparticles that enter the environment are likely to interact with chemical species, which may alter their surface chemistry and interactions with bacteria. To better understand the impact of paired stressors we investigated the impact of subinhibitory Amox exposure in conjunction with IONPs. Initial experiments were carried out to determine the combined impact of functionalized vs. free Amox in the presence of IONPs. Figure 2 outlines the similar impact of IONP and Amox & IONP-Amox conditions on bacterial growth. When exposed to paired stressors (IONP-Amox, and HA & Amox), the bacterial concentration (CFU/mL) and maximum growth rates (μ_max_) obtained were statistically distinguishable from respective individual exposure conditions (Figure 4a,c and Figure 5a,c). Dalgaard et al.’s method was used to calculate μ_max_ values [44]. *S. aureus* and *P. aeruginosa* HA & IONP conditions were run for control purposes, and will not be discussed further. Though not shown, all negative controls consistently lacked bacterial growth and registered OD_600_ values of 0.045 (equivalent to negative control OD_600_ absorbance, containing IONP, Amox, IONP-Amox, and HA).

#### 3.2.1. Bacterial Growth Impacts: IONP-Amox

*S. aureus* & *P. aeruginosa* growth increased when exposed to IONP-Amox. Bacterial concentrations observed in *S. aureus* conditions (*t* = 4, 6 & 24 h) exceeded (*p* < 0.05) those in cultures exposed to Amox or IONP alone, see Figure 4a. Similarly, when exposed to IONP-Amox bacterial concentrations observed in *P. aeruginosa* conditions (*t* = 4 & 6 h), they exceeded (*p* < 0.05) those observed in cultures with only IONP or Amox, see Figure 5a. The μ_max_ for IONP-Amox conditions, in both *S. aureus* and *P. aeruginosa,* also exceeded respective control conditions (*p* < 0.05), see Figure 4c and Figure 5c. 

Increased bacterial concentration (CFU/mL) and μ_max_ seen in *P. aeruginosa* and *S. aureus* cultures exposed to IONP-Amox may be explained by the tendency of subinhibitory antibiotic concentrations to drive an upregulation of iron-dependent cellular components and processes. Antimicrobial agents (i.e., antibiotics) often confer beneficial effects across biological models when low dose exposure occurs, termed hormesis [45,46]. Similarly, the effects of low dose antibiotic impact on maize have been found to show biphasic growth, which is characteristic of hormesis [47]. A recent report by Mathieu et al. provides a detailed account of the pathways by which subinhibitiory antibiotic concentration exposures increase bacterial metabolism and translational capacity [48]. Subinhibitory antibiotic concentrations intensify the uptake and degradation of carbohydrates, nucleosides, and amino acids, while increasing ribosome content and tricarboxylic acid cycle (TCA) activity. Interestingly, nutrient degradation and ribosome synthesis pathways utilize, and at times depend upon, iron [49,50,51]. 

Other researchers have reported increased bacterial growth in the presence of IONPs, paralleling our results. Borcherding et al. report bacterial growth promotion upon exposure to IONP, attributing such growth to particle dissolution and bacterial acquisition [52]. Our data are suggestive of a similar mechanism. The IONP-Amox may have degraded, allowing bacterial acquisition of Amox via cellular porins and iron through Ferric Uptake Regulation (FUR) systems or siderophore production [53,54].

To determine if IONP-Amox underwent Fe ion dissolution, a standard colorimetric detection method based upon the 1,10-phenanthroline response was employed [54,55]. IONP-Amox were allowed to remain in solution for 6 h. After which a reducing agent, hydroxylamine hydrochloride, was added to convert all Fe^3+^ to Fe^2+^. The ferrous ion solution was subsequently treated with 1,10-phenanthroline, which binds Fe^2+^ ions, inducing a solution color change (clear colorless to orange) in iron’s presence. Based on this test, Fe^2+^ ions were present, indicating IONP-Amox dissolution.

From an environmental perspective, the increase in bacterial growth in the presence of IONP-Amox represents an important result. In the environment, bacteria commonly exist within consortia and these results suggest that the addition or removal of limiting resources may destroy otherwise stable bacterial consortia, which contribute to important ecological processes [55,56,57,58,59,60,61,62]. 

These results are also important from a public health perspective given iron’s role in the development of bacterial virulence [63]. In a variety of gram negative and gram positive bacterial models, iron exposure has been found to increase bacterial virulence via altered gene transcription profiles [64]. Literature reports show that an increase in the virulence of environmental bacteria has been shown to increase infections through ingestion and inhalation [65,66].

#### 3.2.2. Bacterial Growth Impacts: HA & Amox

*S. aureus* & *P. aeruginosa* growth increased when exposed to HA & Amox. Bacterial concentrations observed in *S. aureus* conditions (*t* = 6 & 24 h) differed (*p* < 0.05) from those observed in cultures with only HA or Amox (see Figure 4a). The *P. aeruginosa* culture response was similar (see Figure 5a), in that concurrent exposure to HA & Amox produced bacterial concentrations which differed from those of cultures exposed to only HA or Amox. The growth of *P. aeruginosa* cultures exposed to HA exceeded (*p* < 0.05) and differed from cultures exposed to HA & Amox (*t* = 4 & 6 h). *P. aeruginosa* exposed to the HA & Amox exceeded (*p* < 0.05) the Amox alone condition (*t* = 4 & 6 h), but did not statistically differ from the HA alone condition. In terms of μ_max_, HA & Amox-exposed *S. aureus* and *P. aeruginosa* cultures exceeded and differed (*p* < 0.05) from respective control conditions. 

Increased bacterial concentration and μ_max_ seen in both *P. aeruginosa* and *S. aureus* cultures exposed to HA & Amox may be explained by the tendency of subinhibitory Amox concentrations to induce a hormetic response (previously described, Section 3.2.1) in conjunction with HA-induced protective effects. HA has proven capable of protecting bacteria exposed to pollution, drought, ultraviolet radiation, and viral infection [41,42,43]. The mechanism underling HA protective effects is thought to result from pH-specific cell membrane binding and HA metabolism modification [43,67,68]. 

From an environmental perspective, the increase in *P. aeruginosa* and *S. aureus* growth in the presence of HA & Amox is an important result. Environmental bacteria commonly exist within consortia, responsible for cycling elements within the environment. The gradient of available limiting resources over time shapes the structure and function of these assemblages [58,59]. Thus, additional access to limiting resources, via affected metabolism or membrane permeability, and may destroy otherwise stable bacterial consortia, contributing to elemental cycling processes. 

Similarly, these results are important from a public health standpoint because iron exposure has been shown to increase bacterial virulence via altered gene transcription profiles [65,68]. An increase in environmental bacterial virulence has, in select cases, been linked to increased instances of human infection [58].

### 3.3. Impact of all Combined Stressors: IONP-Amox & HA

Given the environmental role played by DOC, it is important to determine how the interaction of IONP-Amox in conjunction with HA, impacts bacterial growth. Therefore, the impact of HA & IONP-Amox was investigated. The HA & IONP-Amox condition was shown to have the greatest impact on bacterial growth. *S. aureus* & *P. aeruginosa* concentrations exceed (*p* < 0.05) all control conditions at each respective time point (save *t* = 0 h), see Figure 4a and Figure 5a. Similarly, HA & IONP-Amox μ_max_ exceeded all respective control conditions (*p* < 0.05) in *S. aureus* and *P. aeruginosa* conditions, see Figure 4c and Figure 5c. 

The increase in *P. aeruginosa* and *S. aureus* growth observed in the presence of HA & IONP-Amox may be due to the combined effects of subinhibitory, antibiotic-induced metabolic upregulation, iron availability, and HA-enhanced metabolism [48,52,67,68]. Studies have shown that subinhibitory antibiotic concentrations upregulate cellular processes responsible for nutrient acquisition [48]. Furthermore, HA has been shown to affect bacterial metabolism through membrane permeability enhancement [67]. Such membrane permeably modification has the potential to facilitate nutrient acquisition, metabolic enhancement, and bacterial growth [67,68]. Taken together, these combined contaminants may enhance bacterial growth.

## 4. Conclusions

Environmental bacteria play a critical role in the maintenance and health of our ecosystems, and their interactions with environmentally transformed nanoparticles are poorly understood. In this work, a study of nanoparticle-bacterial interactions, in conjunction with common pollutants, was conducted as a means of assessing the environmental impact of these entities. 

Our results show that combined nanoparticles, amoxicillin, and DOC enhance bacterial growth. However, individually these substances have little impact on bacteria. From an environmental standpoint, this represents an important result. Assemblages of metabolically intertwined bacterial consortia support the elemental cycling of carbon, nitrogen, sulfur, iron, and oxygen in nature and ensure their consistent availability [5]. The maintenance and stability of these bacterial consortia is rooted in the gradient of available limiting resources present in an environment over time, and the addition or loss of limiting resources, namely iron, may destroy otherwise stable bacterial consortia [5,58,59]. In addition, given the role iron plays in regulation of bacterial virulence, these results hint at the influence iron release could have on public health [63,69,70,71,72,73]. 

While IONPs are often considered to be environmentally friendly and innocuous, our results demonstrate the potential activity of these particles in nature. In combination with low antibiotic concentrations and HA, IONPs may destabilize existing environmental bacterial consortia by increasing their growth and virulence.

## Supplementary Materials

Supplementary materials show the statistical differences in the bacterial growth data. The following are available online at www.mdpi.com/2227-9059/5/3/55/s1.

## References

[1] Lee C., Kim J.Y., Lee W.I., Nelson K.L., Yoon J., Sedlak D.L. (2008). Bactericidal effect of zero-valent iron nanoparticles on *Escherichia coli*. Environ. Sci. Technol..

[2] Morones J.R., Elechiguerra J.L., Camacho A., Holt K., Kouri J.B., Ramírez J.T., Yacaman M.J. (2005). The bactericidal effect of silver nanoparticles. Nanotechnology.

[3] Sondi I., Salopek-Sondi B. (2004). Silver nanoparticles as antimicrobial agent: A case study on *E. coli* as a model for Gram-negative bacteria. J. Colloid Interf. Sci..

[4] Ireland J.C., Klostermann P., Rice E.W., Clark R.M. (1993). Inactivation of Escherichia coli by titanium dioxide photocatalytic oxidation. Appl. Environ. Microbiol..

[5] Knoll A.H., Canfield D.E., Konhauser K.O. (2012). Fundamentals of Geobiology.

[6] Wellington E.M., Boxall A.B., Cross P., Feil E.J., Gaze W.H., Hawkey P.M., Johnson-Rollings A.S., Jones D.L., Lee N.M., Otten W. (2013). The role of the natural environment in the emergence of antibiotic resistance in Gram-negative bacteria. Lancet Infec. Dis..

[7] Linares J.F., Gustafsson I., Baquero F., Martinez J.L. (2006). Antibiotics as intermicrobial signaling agents instead of weapons. Proc. Natl. Acad. Sci. USA.

[8] Goh E.B., Yim G., Tsui W., McClure J., Surette M.G., Davies J. (2002). Transcriptional modulation of bacterial gene expression by subinhibitory concentrations of antibiotics. Proc. Natl. Acad. Sci. USA.

[9] Qiu Z., Yu Y., Chen Z., Jin M., Yang D., Zhao Z., Wang J., Shen Z., Wang X., Qian D. (2012). Nanoalumina promotes the horizontal transfer of multiresistance genes mediated by plasmids across genera. Proc. Natl. Acad. Sci. USA.

[10] Fux C., Costerton J., Stewart P., Stoodley P. (2005). Survival strategies of infectious biofilms. Trends Microbiol..

[11] (2015). The Center for Disease Dynamics Economics and Policy: Global Antibiotic Resistance Partnership, Final Report. Http://cddep.org/publications/state_worlds_antibiotics_2015.

[12] Peng P.C., Wang Y., Liu L.Y., Zou Y.D., Liao X.D., Liang J.B., Wu Y.B. (2016). The excretion and environmental effects of amoxicillin, ciprofloxacin, and doxycycline residues in layer chicken manure. Poult. Sci..

[13] Lienert J., Bürki T., Escher B.I. (2007). Reducing micropollutants with source control: Substance flow analysis of 212 pharmaceuticals in faeces and urine. Water Sci. Technol..

[14] (2013). Executive Agency for Health Consumers: Study on the Environmental Risks of Medicinal Products, Final Report. Https://ec.europa.eu/health//sites/health/files/files/environment/study_environment.pdf.

[15] Landers T.F., Cohen B., Wittum T.E., Larson E.L. (2012). A review of antibiotic use in food animals: Perspective, policy, and potential. Public Health Rep..

[16] Ray P.C., Yu H., Fu P.P. (2009). Toxicity and environmental risks of nanomaterials: Challenges and future needs. J. Environ. Sci. Health C.

[17] Dissanayake N.M., Current K.M., Obare S.O. (2015). Mutagenic effects of Iron oxide nanoparticles on biological cells. Int. J. Mol. Sci..

[18] Nick S.T., Bolandi A., Samuels T.A., Obare S.O. (2014). Advances in understanding the transformation of engineered nanoparticles in the environment. Pure Appl. Chem..

[19] Kapse-Mistry S., Govender T., Srivastava R., Yergeri M. (2014). Nanodrug delivery in reversing multidrug resistance in cancer cells. Front. Pharmacol..

[20] Laurent S., Forge D., Port M., Roch A., Robic C., Vander Elst L., Muller R.N. (2008). Magnetic iron oxide nanoparticles: Synthesis, stabilization, vectorization, physicochemical characterizations, and biological applications. Chem. Rev..

[21] Tepluchin M., Kureti S., Casapu M., Ogel E., Mangold S., Grunwaldt J. (2015). Study on the hydrothermal and SO_2_ stability of Al_2_O_3_-supported manganese and iron oxide catalysts for lean CO oxidation. Catal. Today.

[22] Ghasemi E., Ziyadi H., Afshar A.M., Sillanpää M. (2015). Iron oxide nanofibers: A new magnetic catalyst for azo dyes degradation in aqueous solution. Chem. Eng. J..

[23] Pereira M., Oliveira L., Murad E. (2012). Iron oxide catalysts: Fenton and Fenton-like reactions—A review. Clay Miner..

[24] He F., Li F. (2015). Perovskite promoted iron oxide for hybrid water-splitting and syngas generation with exceptional conversion. Energy Environ. Sci..

[25] Pastrana-Martínez L.M., Pereira N., Lima R., Faria J.L., Gomes H.T., Silva A.M. (2015). Degradation of diphenhydramine by photo-Fenton using magnetically recoverable iron oxide nanoparticles as catalyst. Chem. Eng. J..

[26] Hong Y., Honda R.J., Myung N.V., Walker S.L. (2009). Transport of iron-based nanoparticles: Role of magnetic properties. Environ. Sci. Technol..

[27] Philippe A., Schaumann G.E. (2014). Interactions of dissolved organic matter with natural and engineered inorganic colloids: A review. Environ. Sci. Technol..

[28] Aiken G.R., Hsu-Kim H., Ryan J.N. (2011). Influence of dissolved organic matter on the environmental fate of metals, nanoparticles, and colloids. Environ. Sci. Technol..

[29] Bian S., Mudunkotuwa I.A., Rupasinghe T., Grassian V.H. (2011). Aggregation and dissolution of 4 nm ZnO nanoparticles in aqueous environments: Influence of pH, ionic strength, size, and adsorption of humic acid. Langmuir.

[30] Ma M., Zhang Y., Yu W., Shen H., Zhang H., Gu N. (2003). Preparation and characterization of magnetite nanoparticles coated by amino silane. Colloids Surf. Physicochem. Eng. Asp..

[31] Rehana D., Haleel A.K., Rahiman A.K. (2015). Hydroxy, carboxylic and amino acid functionalized superparamagnetic iron oxide nanoparticles: Synthesis, characterization and in vitro anti-cancer studies. J. Chem. Sci..

[32] Grumezescu A.M., Gestal M.C., Holban A.M., Grumezescu V., Vasile B.Ș., Mogoantă L., Iordache F., Bleotu C., Mogoșanu G.D. (2014). Biocompatible Fe_3_O_4_ increases the efficacy of amoxicillin delivery against Gram-positive and Gram-negative bacteria. Molecules.

[33] Hauser A.K., Mathias R.K., Anderson K.W., Hilt J.Z. (2015). The effects of synthesis method on the physical and chemical properties of dextran coated iron oxide nanoparticles. Mater. Chem. Phys..

[34] Maurer-Jones M.A., Gunsolus I.L., Murphy C.J., Haynes C.L. (2013). Toxicity of engineered nanoparticles in the environment. Anal. Chem..

[35] Brown A.N., Smith K., Samuels T.A., Lu J., Obare S.O., Scott M.E. (2012). Nanoparticles functionalized with ampicillin destroy multiple-antibiotic-resistant isolates of *Pseudomonas aeruginosa* and *Enterobacter aerogenes* and methicillin-resistant *Staphylococcus aureus*. Appl. Environ. Microbiol..

[36] Pirt S.J. (1975). Principles of Microbe and Cell Cultivation.

[37] Collins C.H., Lyne P.M., Grange J.M., Falkinham J.O. (2004). Microbiological Methods.

[38] Nebbioso A., Piccolo A. (2013). Molecular characterization of dissolved organic matter (DOM): A critical review. Anal. Bioanal. Chem..

[39] Pettit R.E. (2004). Organic Matter, Humus, Humate, Humic Acid, Fulvic Acid and Humin: Their Importance in Soil Fertility and Plant Health. https://static1.squarespace.com/static/55c8cff5e4b0af53827c3795/t/56084ddbe4b0da24d92b6e73/1443384795781/Texas+A%26M+Study.pdf.

[40] Fabrega J., Fawcett S.R., Renshaw J.C., Lead J.R. (2009). Silver nanoparticle impact on bacterial growth: Effect of pH, concentration, and organic matter. Environ. Sci. Technol..

[41] Asik B.B., Turan M.A., Celik H., Katkat A.V. (2009). Effects of humic substances on plant growth and mineral nutrients uptake of wheat (Triticum durum cv. Salihli) under conditions of salinity. Asian J. Crop Sci..

[42] Kulikova N., Stepanova E., Koroleva O. (2005). Mitigating activity of humic substances: Direct influence on biota. Use of Humic Substances to Remediate Polluted Environments: From Theory to Practice.

[43] Nardi S., Pizzeghello D., Muscolo A., Vianello A. (2002). Physiological effects of humic substances on higher plants. Soil Biol. Biochem..

[44] Dalgaard P., Koutsoumanis K. (2001). Comparison of maximum specific growth rates and lag times estimated from absorbance and viable count data by different mathematical models. J. Microbiol. Methods.

[45] Calabrese E.J., Baldwin L.A. (2003). Hormesis: The dose-response revolution. Annu. Rev. Pharmacol. Toxicol..

[46] Calabrese E.J. (2005). Paradigm lost, paradigm found: The re-emergence of hormesis as a fundamental dose response model in the toxicological sciences. Environ. Pollut..

[47] Migliore L., Godeas F., De Filippis S.P., Mantovi P., Barchi D., Testa C., Rubattu N., Brambilla G. (2010). Hormetic effect(s) of tetracyclines as environmental contaminant on Zea mays. Environ. Pollut..

[48] Mathieu A., Fleurier S., Frénoy A., Dairou J., Bredeche M., Sanchez-Vizuete P., Song X., Matic I. (2016). Discovery and Function of a General Core Hormetic Stress Response in *E. coli* Induced by Sublethal Concentrations of Antibiotics. Cell Rep..

[49] Robbins A.H., Stout C.D. (1989). Structure of activated aconitase: Formation of the [4Fe-4S] cluster in the crystal. Proc. Natl. Acad. Sci. USA.

[50] Oglesby A.G., Farrow J.M., Lee J.H., Tomaras A.P., Greenberg E.P., Pesci E.C., Vasil M.L. (2008). The influence of iron on *Pseudomonas aeruginosa* physiology: A regulatory link between iron and quorum sensing. J. Biol. Chem..

[51] Somerville G., Mikoryak C.A., Reitzer L. (1999). Physiological characterization of *Pseudomonas aeruginosa* during exotoxin A synthesis: Glutamate, iron limitation, and aconitase activity. J. Bacteriol..

[52] Borcherding J., Baltrusaitis J., Chen H., Stebounova L., Wu C., Rubasinghege G., Mudunkotuwa I.A., Caraballo J.C., Zabner J., Grassian V.H. (2014). Iron oxide nanoparticles induce *Pseudomonas aeruginosa* growth, induce biofilm formation, and inhibit antimicrobial peptide function. Environ. Sci. Nano.

[53] Haley K.P., Skaar E.P. (2012). A battle for iron: Host sequestration and *Staphylococcus aureus* acquisition. Microbes Infect..

[54] Hassett D.J., Sokol P.A., Howell M.L., Ma J.F., Schweizer H.T., Ochsner U., Vasil M.L. (1996). Ferric uptake regulator (Fur) mutants of *Pseudomonas aeruginosa* demonstrate defective siderophore-mediated iron uptake, altered aerobic growth, and decreased superoxide dismutase and catalase activities. J. Bacteriol..

[55] Neilands J. (1972). Evolution of biological iron binding centers. Structure and Bonding.

[56] Hurst C.J. (2007). Neighborhoods and community involvement: No microbe is an island. Manual of Environmental Microbiology.

[57] Behrens S., Kappler A., Obst M. (2012). Linking environmental processes to the in situ functioning of microorganisms by high-resolution secondary ion mass spectrometry (NanoSIMS) and scanning transmission X-ray microscopy (STXM). Environ. Microbiol..

[58] Tilman D. (1985). The resource-ratio hypothesis of plant succession. Am. Nat..

[59] Miller T.E., Burns J.H., Munguia P., Walters E.L., Kneitel J.M., Richards P.M., Mouquet N., Buckley H.L. (2005). A critical review of twenty years’ use of the resource-ratio theory. Am. Nat..

[60] Litwin C.M., Calderwood S.B. (1993). Role of iron in regulation of virulence genes. Clin. Microbiol. Rev..

[61] Messenger A.J., Barclay R. (1983). Bacteria, iron and pathogenicity. Biochem. Educ..

[62] Drakesmith H., Prentice A. (2008). Viral infection and iron metabolism. Nature Rev. Microbiol..

[63] Skaar E.P. (2010). The battle for iron between bacterial pathogens and their vertebrate hosts. PLoS Pathog..

[64] Ratledge C., Dover L.G. (2000). Iron metabolism in pathogenic bacteria. Annu. Rev. Microbiol..

[65] DePaola A., Ulaszek J., Kaysner C.A., Tenge B.J., Nordstrom J.L., Wells J., Puhr N., Gendel S.M. (2003). Molecular, serological, and virulence characteristics of *Vibrio parahaemolyticus* isolated from environmental, food, and clinical sources in North America and Asia. Appl. Environ. Microbiol..

[66] Stewart J.R., Gast R.J., Fujioka R.S., Solo-Gabriele H.M., Meschke J.S., Amaral-Zettler L.A., Del Castillo E., Polz M.F., Collier T.K., Strom M.S. (2008). The coastal environment and human health: Microbial indicators, pathogens, sentinels and reservoirs. Environ. Health.

[67] Vigneault B., Percot A., Lafleur M., Campbell P.G. (2000). Permeability changes in model and phytoplankton membranes in the presence of aquatic humic substances. Environ. Sci. Technol..

[68] Tikhonov V., Yakushev A., Zavgorodnyaya Y.A., Byzov B., Demin V. (2010). Effects of humic acids on the growth of bacteria. Eurasian Soil Sci..

[69] Martinez J., Delgado-Iribarren A., Baquero F. (1990). Mechanisms of iron acquisition and bacterial virulence. FEMS Microbiol. Lett..

[70] Bullen J. (1981). The significance of iron in infection. Rev. Infect. Dis..

[71] Veyrier F.J., Cellier M.F. (2015). Metal economy in host-microbe interactions. Front. Cell. Infect. Microbiol..

[72] Nanotechproject.org. http://www.nanotechproject.org/.

[73] Agrosource.net. www.agrosource.net/pdf/CrisisDeclaration.

