# Supplementary material for: Effect of Iron Oxide Nanoparticles and Amoxicillin on Bacterial Growth in the Presence of Dissolved Organic Carbon
Source: Biomedicines. 2017 Sep 8;5(3):55. doi: 10.3390/biomedicines5030055 (PMC5618313; doi:10.3390/biomedicines5030055)
Supplement: Supplementary file 1 [file biomedicines-05-00055-s001.pdf]

Table S1. Results of statistical analysis for *S. aureus* and *P. aeruginosa* under different experimental conditions at various time points.

S. aureus			*P. aeruginosa*		
Time (h)	Condition	Grouping	Time (h)	Condition	Grouping
0	Positive Control	HI	0	Positive Control	GH
2	Positive Control	HI	2	Positive Control	FGH
4	Positive Control	FGHI	4	Positive Control	DEFG
6	Positive Control	DEFGHI	6	Positive Control	BCDE
24	Positive Control	BCDEFGH	24	Positive Control	BC
0	Amox	HI	0	Amox	GH
2	Amox	HI	2	Amox	GH
4	Amox	FGHI	4	Amox	EFGH
6	Amox	EFGHI	6	Amox	CDE
24	Amox	DEFGHI	24	Amox	BC
0	IONP	HI	0	IONP	GH
2	IONP	HI	2	IONP	GH
4	IONP	FGHI	4	IONP	EFGH
6	IONP	EFGHI	6	IONP	CDE
24	IONP	CDEFGH	24	IONP	BC
0	HA	HI	0	HA	GH
2	HA	HI	2	HA	FGH
4	HA	FGHI	4	HA	EFGH
6	HA	EFGHI	6	HA	CDE
24	HA	HI	24	HA	BCE
0	IONP-Amox	FGHI	0	IONP-Amox	GH
2	IONP-Amox	HI	2	IONP-Amox	GH
4	IONP-Amox	BCDEFGH	4	IONP-Amox	BCDE
6	IONP-Amox	BCDEFGH	6	IONP-Amox	BC
24	IONP-Amox	AB	24	IONP-Amox	BCE
0	HA & IONP	HI	0	HA & IONP	GH
2	HA & IONP	HI	2	HA & IONP	FGH
4	HA & IONP	GHI	4	HA & IONP	EFGH
6	HA & IONP	DEFHGI	6	HA & IONP	BCDE
24	HA & IONP	BCDE	24	HA & IONP	CD
0	HA & Amox	FGHI	0	HA & Amox	GH
2	HA & Amox	FGHI	2	HA & Amox	FGH
4	HA & Amox	FGHI	4	HA & Amox	DEF
6	HA & Amox	DEFGHI	6	HA & Amox	BC
24	HA & Amox	BC	24	HA & Amox	BCE
0	HA & IONP-Amox	HI	0	HA & IONP-Amox	H
2	HA & IONP-Amox	I	2	HA & IONP-Amox	DEFGH
4	HA & IONP-Amox	BCDEF	4	HA & IONP-Amox	A
6	HA & IONP-Amox	BCD	6	HA & IONP-Amox	A
24	HA & IONP-Amox	A	24	HA & IONP-Amox	B

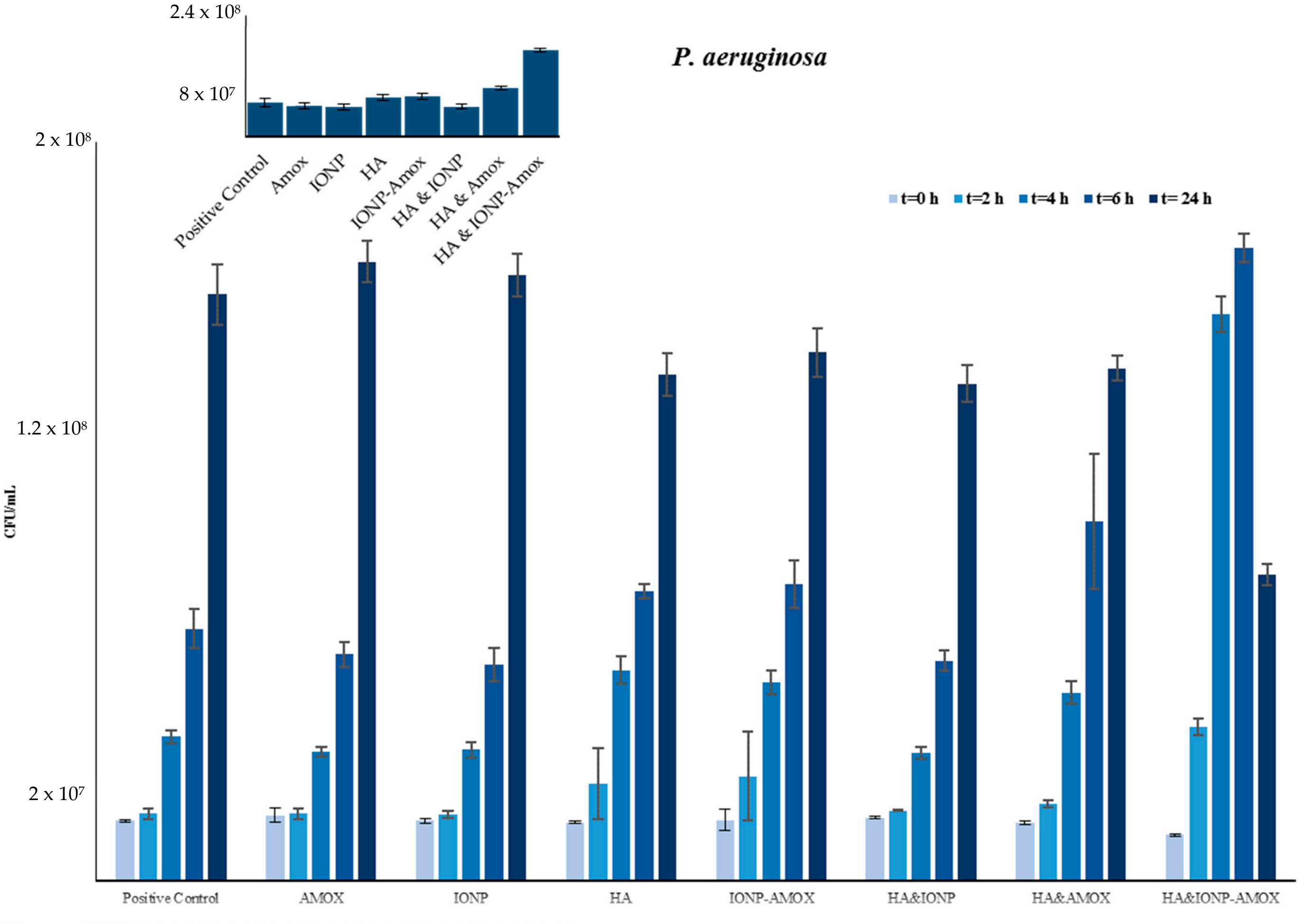

Figure S1. *P. aeruginosa* CFU/mL values for time points 0, 2, 4, 6, & 24 h. Error bars indicate ±SD. [*Inset graph represents t=6 h*]

Comparing the effect on different configurations of IONP and Amox on bacteria

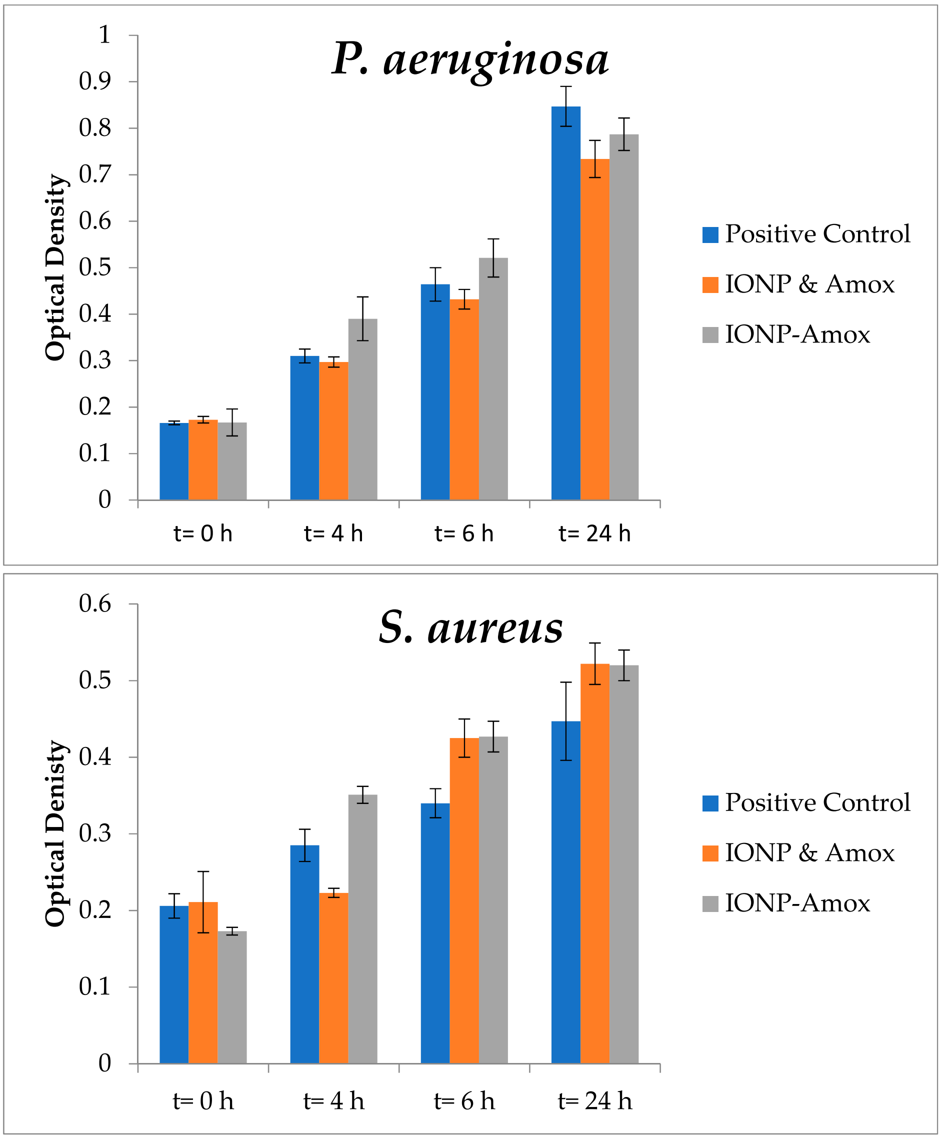

Dash (-) implies functionalization
And (&) implies separate solutions

Figure S2: Bar graph comparing the effect on different configurations of IONP and amox on bacterial growth rates.
